# Understanding metabolic changes in aging bone marrow

**DOI:** 10.1186/s40164-018-0105-x

**Published:** 2018-05-23

**Authors:** Kwasi M. Connor, Young Hsu, Pardeep Kumar Aggarwal, Stephen Capone, Anthony R. Colombo, Giridharan Ramsingh

**Affiliations:** 1 Jane Anne Nohl Division of Hematology and Center for the Study of Blood Diseases, Keck School of Medicine of University of Southern California, 441 Eastlake Ave, MC 9172, Los Angeles, CA 90033 USA; 2 Department of Biology, California Lutheran University, Thousand Oaks, CA 91360 USA

## Abstract

**Background:**

Aging is associated with complex molecular alterations at the cellular level. Bone marrow exhibits distinct phenotypic, genetic and epigenetic alterations with aging. Metabolic changes in the bone marrow related to aging have not been studied.

**Methods:**

In this study, we characterized the metabolome and transcriptome of aging murine bone marrow and compared it with bone marrow from young healthy mice and chemotherapy treated mice; chemotherapy treatment is known to induce age-related changes in hematopoiesis.

**Results:**

The metabolome of the aging bone marrow exhibited a signature of suppressed fatty-acid oxidation: accumulation of free fatty acids, reduced acyl-carnitines and low β-hydroxy butyric acid. The aged bone marrow also exhibited a significant reduction in amino acid and nucleic acid pool. The transcriptome of the aging bone marrow revealed a signature of oxidative stress, known to be associated with mitochondrial dysfunction. Lastly, the metabolic and transcriptomic profiles of the bone marrow of chemotherapy treated mice did not show broad age-related changes but rather mostly resembled young healthy mice, suggestive of a lack of ‘metabolic aging’ with chemotherapy exposure.

**Conclusion:**

Our results revealed broad changes in lipids, amino acids, and nucleotides in aging marrow tissue. Together, these data provide a rich resource for the study of metabolic changes associated with aging in bone marrow.

**Electronic supplementary material:**

The online version of this article (10.1186/s40164-018-0105-x) contains supplementary material, which is available to authorized users.

## Background

Aging is a conserved feature of multicellular organisms and is associated with a gradual decline in cellular, tissue, and organ function. The molecular mechanisms of aging can be attributed to DNA damage, epigenetic shifts, and altered RNA and protein profiles [1]. Collectively, these changes may increase the likelihood of many age-related diseases. Hence, understanding the underlying mechanisms of aging is imperative for preventing and developing treatment for age-related illnesses. While efforts to understand the underlying mechanistic changes of aging have focused primarily on RNA and DNA alterations, fewer studies have investigated the metabolomics of aging.

The hematopoietic system undergoes significant functional changes with aging, including reduced self-renewal and an increased myeloid-skewing of hematopoietic stem cells (HSCs), immunodeficiency, loss of cellularity, and increased deposition of fat cells in the bone marrow [2]. Bone marrow is a heterogeneous tissue that is responsible for the production of diverse blood cells over the lifetime of the individual. The aging hematopoietic system increases the risk of many hematological disorders, such as anemia, myelodysplastic syndrome (MDS), and leukemia. Significant age-related molecular changes in DNA and RNA of specific cell types of the bone marrow have been described. For example, DNA damage and telomere shortening has been shown to occur in aged HSCs and hematopoietic progenitors [3–5]. These studies have provided significant advancement to understanding the aging hematopoietic system. However, global molecular and biochemical characterization of bulkbone marrow as a tissue can provide additional clarity to the understanding of mechanisms that govern hematopoietic aging.

Chemotherapy has been shown to increase premature age-related health problems, such as coronary heart disease, cognitive decline and osteoporosis, and poses long-term risks of secondary cancers including MDS, and acute myeloid leukemia (AML) [6–11]. Direct in vivo studies have also shown that the effect of chemotherapy mimics several age-related changes in hematopoiesis [6–8]. In light of these findings, we wanted to understand whether chemotherapy alters the metabolic profile of bone marrow and whether these resemble changes associated with aging.

Current metabolomic approaches provide a robust global analysis of tissue-related changes that occur with exposure to external agents, environmental shifts, or the passage of time. A limited number of studies have investigated the metabolome in aging models and have shown significant alterations in lipid and amino acid metabolism [9, 10]. However, no study has characterized the metabolome of aging bone marrow. In the current study, we characterized the metabolomic alterations in murine bone marrow with aging and following exposure to chemotherapy. To understand the molecular mechanisms underlying the observed metabolic changes, we measured the transcriptome-wide expression of genes using RNA-sequencing. These approaches allowed for a comprehensive analysis of the overlap between aging and chemotherapy-related changes to bone marrow cells in vivo.

## Methods

### Murine treatment and bone marrow isolation

Twenty 8-week old and eleven 72-week old male BALB/c mice were used for the experiment. 8-week old mice were injected with 0.15 mg/g of cyclophosphamide (chemotherapy group, N = 10) or saline (young group, N = 10) intraperitoneally every 28 days, for a total of three cycles. During the same period, the 72-week old mice (N = 11) were observed in similar vivarium conditions. 28 days after the last chemotherapy/saline treatment (young and chemo group were 24 weeks old and old group was 88 weeks old), all mice were sacrificed (after 16 h of fasting). One chemotherapy-treated mouse died before bone-marrow extraction. Tibia and femur bones were flushed with PBS containing 5% FBS. Spines were crushed with mortar and pestle in PBS with 5% FBS. The cell suspensions were combined and strained (5 μm). Subsequently, red blood cells were depleted using red blood cell lysis buffer (Sigma-Aldrich; Saint Luis, MO) and centrifuged at 300 G. A 200 μl aliquot of the cells in suspension were used for RNA extraction and the remaining cells were used for the metabolomic assay. For the metabolomic analysis, the bone marrow cells were pelleted (25 μl), snap frozen and stored at − 80 °C until the assay was performed. The animal protocol was approved by the Institutional Animal Care and Use Committee (#11913) at University of Southern California, and all the experiments were done in accordance with the guidelines and regulations mandated by the committee.

### Metabolomic assay

Frozen samples were shipped on dry ice to Metabolon (Durham, NC), where the assay was performed. Metabolites were measured according to published protocols [11]. Briefly, samples were homogenized and subjected to methanol extraction, then split into aliquots for analysis by ultrahigh performance liquid chromatography/mass spectrometry (UHPLC/MS) in the positive, negative, or polar ion mode. Metabolites were identified by automated comparison of ion features to a reference library of chemical standards followed by visual inspection for quality control (as previously described [12]). Raw ion-intensities for each biochemical were detected on a per sample basis (Additional file 1: Table S1) followed by median rescaling, such that each biochemical median across all samples is equal to 1. Missing values were imputed with the lowest observed value in the dataset. The assumption with this approach is that the missing biochemicals represent metabolites that fell below the limit of detection, rather than not being present at all. This approach preserves the biological variation and promotes more accessible data presentation. Each sample underwent normalization by the median of the rescaled raw ion-intensities of all metabolites measured per sample. *Statistical analysis*: A one-way ANOVA followed by Tukey’s test was used to test the null hypothesis of no difference in the mean values of each metabolite between experimental-groups. A *P* < 0.05 was used as the criteria to reject the null hypothesis in each analysis. IBM SPSS Statistics™ ver. 24 was used to perform ANOVA and Tukey analyses. Outlier detection was used to identify trends in certain cases, by using a median-based technique in R-package Rallfun-v27 and RStudio 1.0.44.

Metabolite pathway association analysis of lipids, amino acids, and nucleotides was performed using a formula derived from Metabolome’s Metabolync ™ software—(k/m)/(n/N); k = differentially expressed metabolite in pathway; m = detected metabolites in a pathway; n = all differentially expressed metabolites; N = all detected metabolites. The calculated expression ratios allowed for a rank of human metabolome database (HMDB) pathways associated with the differentially expressed metabolites between treatment groups.

### RNA sequencing

Three samples from each experimental-group were used for RNA-sequencing. RNA was isolated from frozen bone marrow using GeneJET Whole Blood RNA Purification Mini Kit (Thermo Scientific; Waltham, MA) following the manufacturer’s protocols. 250 nanograms of RNA was used to generate RNA-sequencing libraries using the TruSeq Stranded mRNA library prep kit (Illumina; San Diego, CA) according to manufacturer’s protocols. Sequencing was performed on an Illumina NextSeq500 system that produced paired-end 75 bp reads—an estimated 33 million reads per sample. The RNA-seq reads were quantified using Arkas [13] which quantified abundance estimates and assembled the quantification data annotating against ENSEMBL build 88 coding and non-coding genes. *Statistical analysis:* Pair-wise-differential expression between treatment groups (aged and chemotherapy-treated) and control (young) was determined with a limma-based general linear model using Edge-R software. Genes with a *P* < 0.05 and *FDR* < 0.05 were considered differentially expressed. Pathway analysis was performed using ingenuity pathway analysis (IPA). Statistically significant pathways were identified using a Fisher’s exact test. Pathways with a *P* < 0.05 were considered significantly activated. IPA was also used to predict regulator genes and their downstream target molecules that were differentially expressed in our dataset.

### Flow cytometry

24 months old (aged) and 5 months old (young) Balb/c mice were euthanized and bone marrow was aspirated from tibia and femur using PBS + 0.5% bovine serum albumin (BSA) + 2 mM EDTA. After 5 min incubation in RBC lysis buffer, the mononuclear cells were washed with PBS and re-suspended in 0.5% BSA + DPBS + 2 mM EDTA. The cells were then incubated for 30 min at 4 °C with monoclonal antibodies CD45, CD3e, CD45R (B220), MAC-1(CD11/b) from eBiosciences and TER-119 (Biolegend) and washed with DPBS + 2 mM EDTA. The flow cytometry was performed using BD FACS Verse and analyzed using FlowJo v10 analysis software.

## Results

### Global alterations in the metabolome of aging and chemotherapy treated bone marrow

**Fig. 1:**
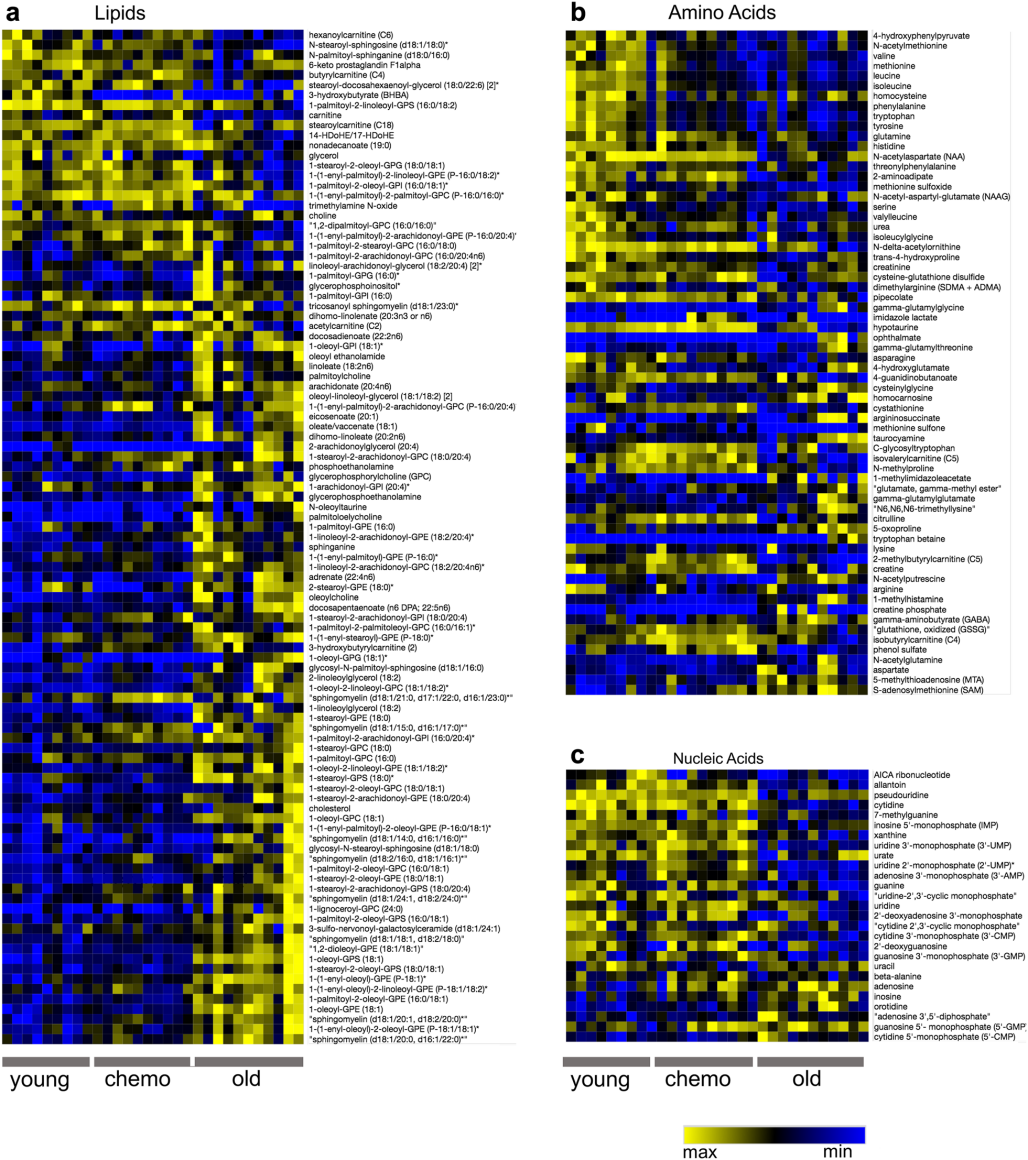
Relative abundances of dysregulated metabolites identified by ANOVA. Metabolites were ordered by ranked list (Pearson’s correlation). Yellow or blue color reflects higher and lower Log2 expression levels respectively. **a** Lipids. **b** Amino acids. **c** Nucleic acids. The corresponding Log2 max and min values are as follows; Lipids (2.7, − 2.2), Amino Acids (4.0, − 4.1), (2.7, − 3.9)

Metabolon assay using UHPLC/MS identified 450 metabolic signatures across samples. 438 metabolites were related to lipids, amino acids, nucleotides, carbohydrates, energy-related biochemicals (i.e. TCA cycle, electron transport chain), vitamins, and co-factors. ANOVA revealed 211 significantly differentially expressed (i.e. dysregulated) metabolites between young, old, and chemotherapy-treated mice (Additional file 2: Table S2) (of the 211 significantly differentially expressed metabolites, 199 passed the Tukey’s test). Of these, a total of 101, 66, and 27, lipid, amino acid, and nucleotide metabolites respectively were differentially expressed between treatment groups (Fig. 1a–c, Additional file 2: Table S2). Also, 7 carbohydrates and 10 vitamins/co-factors were differentially expressed between treatment groups (Additional file 2: Table S2). No biochemical represented by the TCA cycle or electron transport chain was differentially expressed between treatment groups.

### Concordance between aging and chemotherapy-exposed bone marrow

**Fig. 2:**
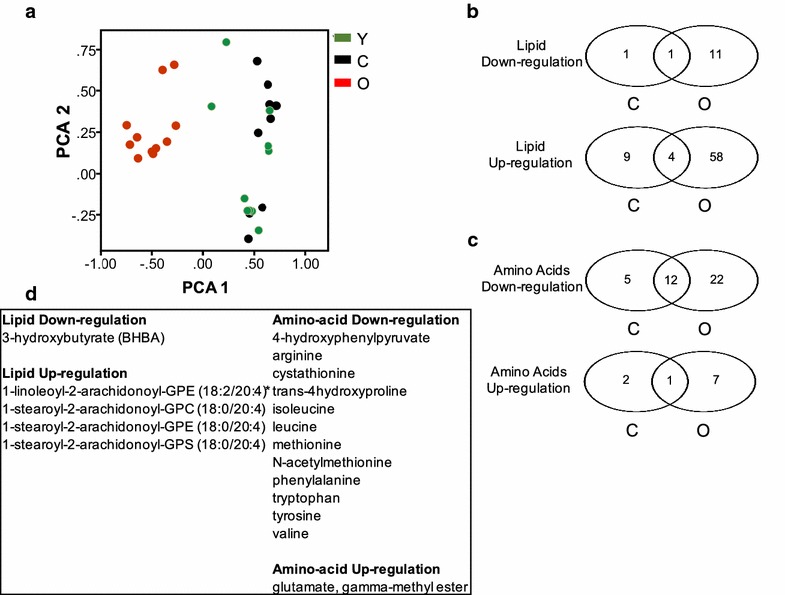
Metabolomic comparisons between experimental groups. **a** PCA analysis of the expression of 211 dysregulated (ANOVA) metabolites per sample-group. **b** Overlap of lipids and **c** amino acids dysregulated (ANOVA) between experimental groups. **d** List of the metabolites that overlap between old and chemotherapy-treated mice

PCA using the 211 dysregulated metabolites revealed two relevant components. A plot of the components of the differentially expressed metabolites revealed a close association between young and chemotherapy-treated mice (Fig. 2a); Kaiser–Meyer–Olkin adequacy = 0.76, Bartlett’s test of sphericity < 0.001.

Tukey’s tests identified a higher number of metabolites up- or down-regulated in the old (compared to young) than the chemotherapy-treated mice (compared to young); 74, 43, and 17, lipid, amino acid and nucleotide metabolites, respectively were differentially expressed in old (Additional file 2: Table S2). In comparison, 15, 20, and 6, lipid, amino acid, and nucleotide metabolites, were differentially expressed respectively in chemotherapy-treated mice (Additional file 2: Table S2). Among the Tukey test-identified differentially expressed lipid metabolites, only five overlapped between old and chemotherapy-treated mice (Fig. 2b, Additional file 2: Table S2). Similarly, a total of 12 amino acid metabolites overlapped between these treatment-groups (Fig. 2c; Additional file 2: Table S2). Of these 12 overlapping metabolites, 11 were down-regulated (no alteration of a particular sub-pathway was identified in this group) and 1 up-regulated (glutamate, gamma-methyl ester). Only three differentially-expressed nucleic-acid metabolites overlapped between the treatment groups (Additional file 2: Table S2). No overlaps occurred between the treatment groups in the expression of carbohydrates, vitamins, and cofactors (Additional file 2: Table S2). Together, these data suggest that the compendium of dysregulated metabolites of old marrow differed markedly from the young marrow. The bone marrow of chemotherapy-treated mice had a more similar metabolic profile with the young marrow than the old marrow.

### Altered biochemical pathways in aging bone marrow

**Fig. 3:**
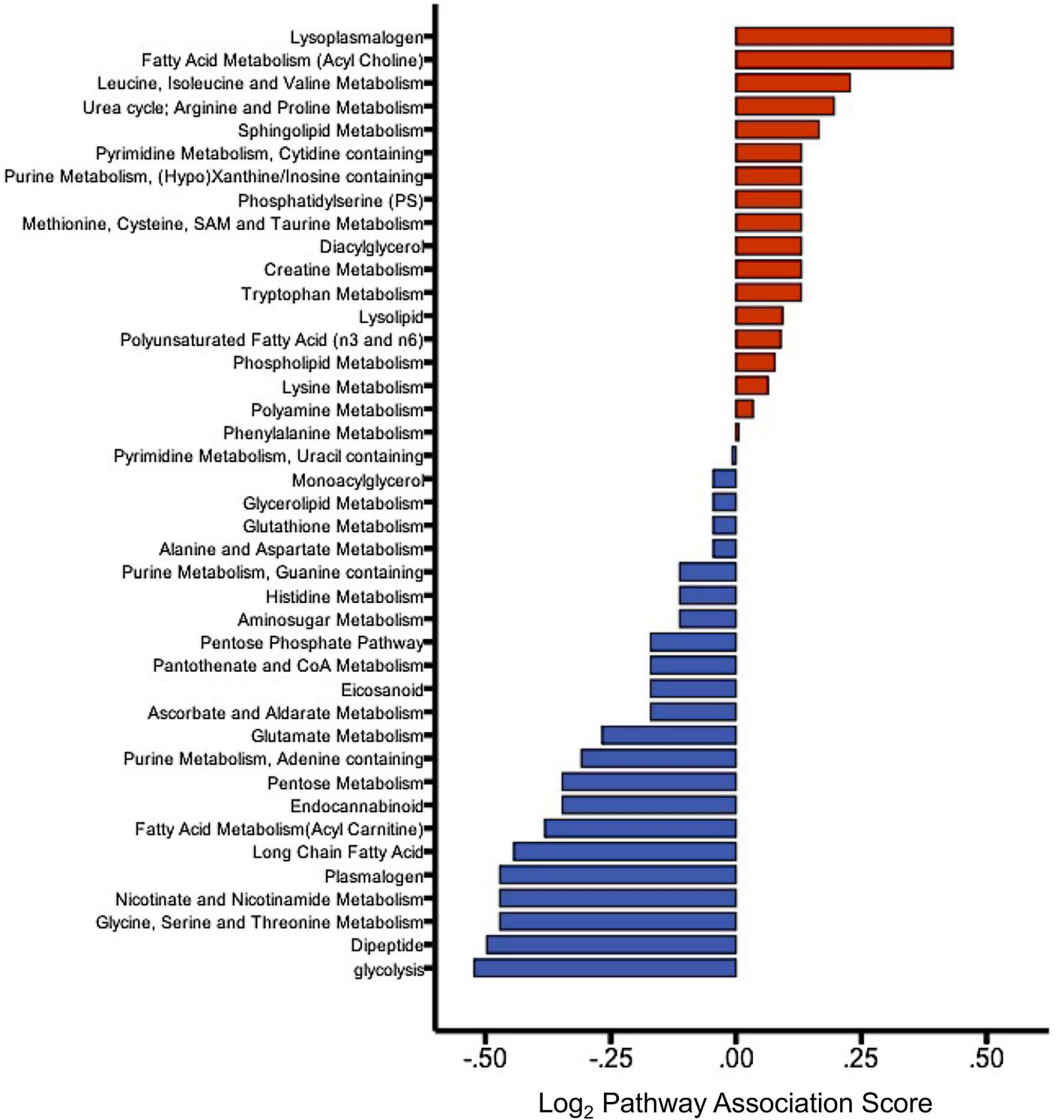
Rank of pathways associated with aging. Red or blue color reflects positive (up-regulated) and negative (down-regulated) association-scores

In agreement with other studies of aging tissues [9, 10], lipids were enriched in old bone marrow including the sub-classes lysoplasmogens, lysolipids, phospholipids, polyunsaturated fatty-acids, long-chain fatty-acids, monoacylglycerols, sterols, sphingolipids, diacylglycerols, and an endocannabinoid. Amino acids were diminished in old mice potentially affecting energetic and anabolic potential. Interestingly, these amino acids were highly composed of essential and non-essential amino acids, including valine, histadine, methionine, leucine, isoleucine, phenylalanine, tryptophan, tyrosine, and glutamine (Additional file 2: Table S2). Furthermore, 17/20 differentially expressed amino acids in chemotherapy-treated mice were down-regulated (Additional file 1: Table S1). Our pathway association analysis of the 211 significantly differentially expressed metabolites in old mice revealed which pathways were most affected by aging. The top three up-regulated pathways in aging were ‘lysoplasmogen’, ‘fatty-acid metabolism (acyl-choline)’, and ‘leucine, isoleucine and valine metabolism’. The top three down-regulated pathways in aging were ‘glycolysis’, ‘dipeptide’ and ‘glycine, serine and threonine metabolism’ (Fig. 3).

### Specific alterations of aging and chemotherapy-exposed bone marrow

A number of specific metabolites dysregulated in old and chemotherapy-treated mice were noteworthy. Lipids: In agreement with lowered fatty-acid oxidation with age, the ketone body, 3-hydroxybutyrate (BHBA) was > twofold lower (*hypoketosis*) in old mice and also robustly lowered in chemotherapy-treated mice (Additional file 2: Table S2). 7 out of 8 differentially expressed (compared to young) phospholipids were up-regulated in chemo-treated mice (Additional file 2: Table S2). Amino Acids: the expression of methionine pathway differed between groups. Notably, methionine, cystathionine, methionine sulfone, and methionine sulfoxide were down-regulated in old marrow. S-adenosylmethionine (SAM), which is a key contributor of methyl groups to the epigenome, was up-regulated (1.56 fc) (Additional file 2: Table S2). Furthermore, molecules related to GSH synthesis were up-regulated in old mice. ANOVA identified γ**-**glutamyl-glutamate, γ-glutamyl-threonine, 5-oxoproline, cysteinyl-glycine as up-regulated metabolites in old mice (Additional file 2: Table S2), in line with the well-known positive association between aging and oxidative stress [14]. Strikingly, 17 out of 20 differently expressed amino acids dysregulated (Tukey’s test) in chemotherapy-treated mice were down-regulated (Additional file 2: Table S2). Nucleic acids: Interestingly, 3 uridine derivatives were down-regulated in old-marrow, in agreement with a temporal-based study that revealed a gradual decline of uridine in aging mouse liver tissue [15] (Additional file 2: Table S2).

We also analyzed pathways related to energy-state. β-oxidation of free fatty acids (FFAs) (fatty-acid oxidation), a process by which FFAs are transported across the mitochondrial membrane and converted to acetyl-coA for energy production, appeared disrupted in old bone marrow. ANOVA identified the accumulation of several classes of lipids related to fatty-acid oxidation (FAO) including 2/3 LCFAs, 3/3 monoglycerols, 2/3 diaglycerols and 7/7 PUFAs (11 of these up-regulated lipids were confirmed by Tukey’s test) (Additional file 2: Table S2). ANOVA also identified 4/5 fatty-acyl-carnitines down-regulated in old bone marrow (2 of these down-regulated fatty-acyl-carnitines were confirmed by Tukey’s test) (Additional file 2: Table S2). Furthermore, carnitine was significantly down-regulated in old bone marrow (Additional file 2: Table S2). Finally, following the removal of one outlier, lactate was elevated in old marrow in agreement with bio-energetic signatures of previously observed aging models [9, 16].

### Transcriptome changes with aging and chemotherapy exposure in bone marrow

**Fig. 4:**
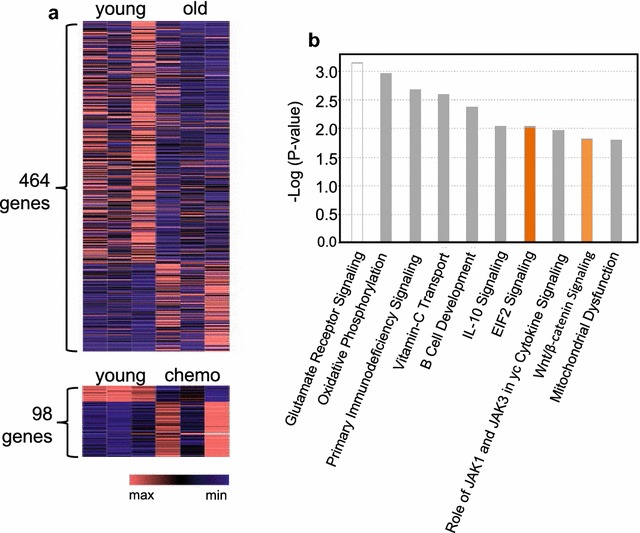
Gene expression in old and chemotherapy. **a** Heatmap of pair-wise analysis of differential (limma-based general linear model) gene expression (log2 (1 + x) counts per million); YvsO and YvsC. The corresponding max and min values are as follows; YvsO (0, 12.96), YvsC (0, 13.52). **b** IPA core-pathway analysis; no color = no activity detected; grey color = direction of activity cannot be predicted; orange color = up-regulated activity. Color intensity reflects level of expression

A limma-based general linear model, pair-wise analysis revealed 464 and 98 differentially expressed genes between old vs. young and chemotherapy-treated vs. young pairs, respectively Fig. (4a). The old vs. young comparison revealed 340 up-regulated and 124 down-regulated genes in old mice marrow (Fig. 4a, Additional file 3: Table S3). The chemotherapy-treated vs. young comparison revealed 22 up-regulated and 76 down-regulated genes in chemotherapy-treated mice (Fig. 4a, Additional file 4: Table S4). The smaller gene list that resulted from the chemotherapy-treated analysis is representative of greater similarity in gene expression between young and chemotherapy-treated mice than young and old.

IPA analysis revealed a number of pathways dysregulated in aging marrow that are associated with stress and dysfunction within the bone marrow compartment, such as ‘B Cell Development’, ‘EIF2 Signaling’, ‘Wnt-β-catenin’ and ‘Mitochondrial Dysfunction’ (Fig. 4b, Additional file 5: Table S5). Furthermore, up-stream analysis predicted the activation of a number of regulatory-genes in old marrow associated with inflammation, such as interleukin-7, tumor necrosis factor and interferon gamma. Lastly, IPA predicted pathological states in old-marrow tissue including decreased ‘quantity of leukocytes and lymphocytes’ suggestive of myeloid skewing at the molecular level.

## Discussion

Our study was the first to comprehensively characterize the metabolic alterations in murine bone marrow. It revealed considerable age-related changes, with profound alterations in the composition of lipid, amino acid and nucleic acid metabolites. Aging bone marrow showed pronounced accumulation of FFAs, and a diminished amino acid and nucleic acid pool. Remarkably, these changes were similar to the changes described in aging nematodes [17]. A number of PUFAs, LCFAs, mono-and di-acylglycerols were up-regulated in aged bone marrow. Interestingly, acyl-carnitines, which are FAO intermediates, were down-regulated. These findings are suggestive of reduced “FAO flux” observed in aged mouse blood [9]. Fatty acids are conjugated to carnitine molecules which allow them to pass through the inner mitochondrial membrane where they are converted to fatty acyl-CoA molecules that enter the TCA cycle for energy production. Our finding of elevated FFAs and decreased carnitine-conjugates is strong evidence of suppressed β-oxidation [9, 18]. Supportive of this inference, we observed a down-regulation of genes involved in β-oxidation such as Acyl-CoA synthatases *Acss3* and *Acss2*. Furthermore, the ketone body 3-hydroxybutyrate (BHBA), which is known to correlate with the level of fatty acid oxidation, was also down-regulated in old mice [19].

Perturbations of the β-oxidation pathway are known to occur by way of intrinsic (biological aging) and extrinsic factors (toxins) [20–22]. We found an up-regulation of several mitochondrial electron transport genes in the transcriptome of old bone marrow in agreement with increased expression of electron transport genes of mtDNA with aging [23, 24]. Disruptions of the electron transport chain in mitochondria can generate reactive oxygen species (ROS), which in turn may cause further deterioration of mitochondrial function, generating an amplifying feedback loop [22].

Activation of the ‘oxidative phosphorylation’ pathway, which was observed in the aged bone marrow, is a major source of ROS production. Furthermore, the metabolic and molecular signatures of the old marrow showed features that suggest an activation of compensatory mechanisms to oxidative stress, a feature known to be associated with aging in other mammalian tissues [25–29]. Strikingly, ophthalmic acid, which is an endogenous antioxidant synthesized during episodes of oxidative stress [30], was ≈ ninefold higher in old mice. Although the metabolite GHS itself was not altered in old mice, cysteine-glutathione disulfide which is as a prodrug of GHS [31], appeared down-regulated in old mice. Correspondingly, the metabolites involved in the synthesis of the antioxidants (γ-glutamyl-glycine, γ-glutamyl-glutamate, γ-glutamyl-threonine, 5-oxoproline, cysteinyl-glycine) [14] were elevated in aged bone marrow. Although the metabolites involved in the anti-oxidant pathway appeared up-regulated in the aged bone marrow, it is possible that their elevation may not be sufficient to compensate for the level of oxidative stress in the aged cell. It remains unclear how these observed complex metabolic alterations in aged bone marrow are mechanistically linked to each other. It is likely that they influence each other, leading to a cumulative effect that contributes to age-related functional decline. Interestingly, phospholipids, lysolipids and lysoplasmalogens (lysolipids and lysoplasmalogens are intermediate products of phospholipid pathway) were also robustly elevated in old mice. Enhanced production of ROS can lead to peroxidation of phospholipids, which can possibly affect their normal recycling pattern [32].

Bone marrow of old mice exhibited broad reductions in amino acid metabolite levels. Amino acids in excess of metabolic demand are rapidly degraded in cells, rather than stored. Hence, snapshot measurements of amino acids reflect an “amino acid flux-state”. Our finding of a diminished amino acid pool in aged bone marrow is in agreement with studies of aging rat brain tissue [10, 33] and aging mammalian serum [9, 10]. A diminished amino acid pool in aging could be the result of reduced synthesis of non-essential amino acids or from lowered autophagy [34, 35]. Autophagy, which replenishes the amino acid pool via protein turnover, has been shown to be depressed with aging [35]. Furthermore, intracellular availability of amino acids can directly affect the rate of protein synthesis [36]. We found up-regulation of EIF2 signaling pathway in the transcriptome of old bone marrow. The activation of EIF2 signaling has been shown to play a compensatory role in amino acid deficiency state [36]. However, comprehensive mechanistic studies of amino-acid deficiency in aging and its relationship to EIF2 signaling are needed to determine a direct link between these processes.

Other metabolites showed signs of deterioration in aged marrow. For example, nucleic acids were down-regulated in old marrow. This may partially be a result of lipid peroxidation, which has been shown to generate hydroperoxides that undergo fragmentation to produce a broad range of intermediates called reactive carbonyl species (RCS). RCS can then react with nucleophilic groups in proteins, DNA and aminophospholipids to form adducts that are subsequently degraded. Also, peroxidation-derived end-products can also react on the exocyclic amino groups of deoxyguanosine, deoxyadenosine [32], which were down-regulated in aged bone marrow. We did not see broad changes in carbohydrate metabolism between the groups. However, lactate was elevated in old marrow suggestive of a relative increase in anaerobic glycolysis, which is in agreement with previous studies of aging tissue [9].

Our analysis of the transcriptome of the old bone marrow exhibited an inflammatory signature, a known feature of aging. The top three upstream molecules identified by IPA analysis were cytokines, including interleukin 7, tumor necrosis factor, and interferon-gamma, which target a total of 97 molecules differentially expressed in our dataset. Furthermore, the Wnt-β-catenin pathway was down-regulated in the transcriptome of the old bone marrow. Wnt signaling plays a major role in hematopoiesis [37]. In agreement with these findings and previous studies of immunosenescence, the ‘B cell development pathway’, including the genes *RAG1* and *RAG2*, which initiate V(D)J recombination in B-cells, was down-regulated in aged bone marrow.

**Fig. 5:**
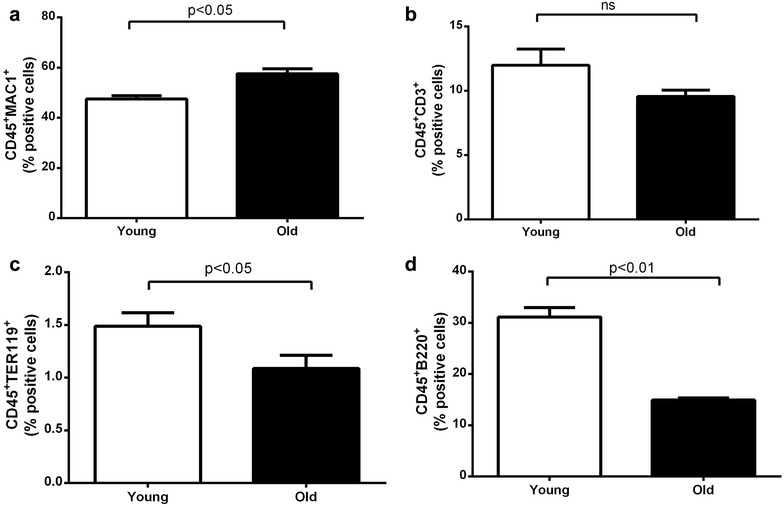
Bonemarrow cellular composition comparing old and young mice. **a** MAC1+ve leucocytes (Myeloid cells). **b** CD+ve cells (T-cells). **c** Ter119+ve cells (erythroid cells). **d** B220+ve cells (B-cells). P value for statistical difference was calculated using paired t test

Aging is also associated with alterations in cellular composition in the bone marrow [38]. Our analysis comparing the cellular composition of young and old bone marrow showed significant changes with aging (Fig. 5). Hence the metabolic and transcriptomic changes may reflect both the alterations in cellular compartment and intrinsic changes within each cellular compartment. In order to understand the relative contribution from aging at cellular level and changes in cellular composition in the bone marrow with aging, future metabolomic analysis of individual cellular components of the bone marrow would be necessary.

Finally, our study did not show a significant overlap between age-related and chemotherapy-related changes in the metabolome or the transcriptome of murine bone marrow. However, a compendium of amino acids was diminished in chemotherapy-treated mice, a feature seen in aged marrow. A prior study by Beerman et al. analyzed chemotherapy-related methylation alterations in the murine HSCs and showed that chemotherapy exposure recapitulated several age-related changes [6]. Our study examined the entire bone marrow compartment, which is largely comprised of progenitors and differentiated cells. It is possible that the age-related alterations seen with chemotherapy exposure are restricted to HSCs and early progenitors. Toward this end, Wang et al. [39] suggested that residual bone marrow injury following chemotherapy is primarily a consequence of intrinsic damage to HSCs that leads to senescence. This was unlikely to be captured by our analysis that examined the entire bone marrow. Also, it is unknown if molecular and biochemical changes in HSCs will be reflected in their progeny, which makes up the majority of the bone marrow. It is also possible that the HSCs that exhibit significant chemotherapy-related alterations would fail to differentiate, with the differentiated cells arising from the increased levels of healthy HSC counterparts, and hence the lack of chemotherapy-related effects reflected in the bulk-bone marrow. Age-related changes induced by chemotherapy could also be related to the specific chemotherapy agent used. The previous study by Beerman et al. [6] that showed age-related methylation changes with chemotherapy exposure used 5-fluorouracil. 5-fluorouracil primarily targets the progenitors as opposed to cyclophosphamide, which is an alkylator known to induce DNA damage directly in HSC [39].

In summary, our comprehensive characterization of the metabolome and transcriptome of aging murine bone marrow shows changes suggestive of a significant shift in energetic and anabolic state. Our global approach to understanding the aging bone marrow provides a tractable resource for future research that dials-down on the refined mechanistic causes and consequence of metabolic alterations seen in aging bone marrow. Particularly, studies of the role of mitochondrial dysfunction and its metabolic consequences in human aging and age-related hematological diseases are warranted.

## Additional files

**Additional file 1: Table S1.** Mouse metabolome: raw ion-intensities for each biochemical were detected on a per sample basis..

**Additional file 2: Table S2.** One-way ANOVA of metabolite expression, followed by post hoc Tukey tests across treatment groups.

**Additional file 3: Table S3.** RNA-seq gene expression in the marrow of old mice.

**Additional file 4: Table S4.** RNA-seq gene expression in the marrow of chemo-treated mice.

**Additional file 5: Table S5.** IPA pathway analysis of old-mice marrow.
